# Exploring Ghanaian adolescents’ meaning of health and wellbeing: A psychosocial perspective

**DOI:** 10.3402/qhw.v10.26370

**Published:** 2015-04-07

**Authors:** Franklin N. Glozah

**Affiliations:** 1 Department of Psychology and Human Development, Regent University College of Science and Technology, Accra, Ghana; 2 School of Health and Human Sciences, University of Essex, Colchester, UK

**Keywords:** Social support, stress, health and wellbeing, social constructionism, qualitative method, thematic analysis, adolescents, Ghana

## Abstract

There is presently no internationally agreed upon set of indicators for assessing adolescent health and what “health and wellbeing” means to adolescents. The psychosocial context of family, friends, and school plays a crucial role in the construction of health and wellbeing by adolescents. In spite of this, not much is known about the meaning Ghanaian adolescents attach to their health and wellbeing and the role of stress and social support in the construction of this meaning. This study explores how perceived social support and stress influence the construction of the meaning of health and wellbeing to Ghanaian adolescents. Eleven respondents purposively selected from 770 males and females participated in semi-structured interviews, which were transcribed verbatim and analysed with thematic analysis. Findings pointed to the fact that health and wellbeing was largely construed as “ability to perform daily functions,” such as ability to take critical decisions and a general sense of vitality and mental strength. These were influenced by perceived social support (“encouragement and advice” and “religiosity or spirituality”) and stress (“teasing, strictness, quarrels, and arguments”). These findings suggest that effective communication, mutual respect, and support from significant others, in the midst of stressful life events, contribute substantially to a holistic construction and meaning of health and wellbeing by Ghanaian adolescents.

Children and adolescents are usually regarded as healthy but every year an estimated 1.7 million adolescents lose their lives mostly through substance abuse, malnutrition/poor eating habits, lack of physical activity, suicide, violence, illnesses, and other physical and mental health disorders (WHO, 2008). As such, standardized frameworks for health indicators in young people are available for some high-income countries with a few producing regular reports on the health status of adolescents (Starfield, Bergner, Ensminger et al., 1993). Ghana is presently undergoing rapid social and economic changes, and these can have a profound effect on adolescents, in relation to their health and wellbeing and experience of social support and stressful life events. It is known that social and economic policies have an association with physiological changes in children and/or their close family members (Berkman & O'Donnell, 2013).

At present, there is no internationally agreed upon set of indicators for assessing adolescent health (Patton et al., 2012). The Millennium Development Goals have adopted some indicators of the development of young people in low- and middle-income countries, but with a focus predominantly on sexual and reproductive health (Beaglehole & Bonita, 2008). This is the situation currently in Ghana with adolescent sexual and reproductive health status as the main focus (e.g., Ahiadeke, 2005; Awusabo-Asare, Biddlecom, Kumi-Kyereme, & Patterson, 2006). For adolescents there is a need for indicators to go beyond sexual and reproductive health as more comprehensive approaches for assessing adolescent health status would outline relevant social determinants of health as well as the contribution of adolescent-onset risk states and behaviours to future disease burden (Walker, Bryce, & Black, 2008). Indeed, the health status reports on adolescents have progressed from an earlier focus on age differentials of regularly collected data, to the more recent inclusion of non-communicable diseases as well as contextual and social determinants of health (UNICEF, 2007).

The concept of wellbeing has a multidimensional constitution—it could be a representation of positive feelings individuals experience as well as aspects of life characterized by optimal functioning and flourishing (Fredrickson & Losada, 2005). It has been asserted that it is practical to assume that the concept of health is comparable to the concept of wellbeing (Essen & Martensson, 2014). Accordingly, “health and wellbeing” has been used in the present study to represent a composite concept in accordance with the WHO (1947) definition of health as: “a state of complete physical, psychological, and social wellbeing and not only the absence of disease or infirmities.”

This study utilizes a social constructionist epistemology to understand the idea that reality can only be understood in relation to the beliefs, thoughts, and perceptions of an individual (Gergen, 1985). The aim of social constructionism is to redefine commonly used psychological constructs (i.e., mind, self, and emotion) as socially constructed, rather than individually constructed processes. According to the social constructionist view, the generation of knowledge and ideas of reality are not sparked by individuals but through social processes (Gergen, 1994). With its emphasis on individualization, participation, self-determination, human rights, and social justice, a social constructionist perspective, in terms of social processes and networking, may be directly or indirectly associated with individual wellbeing by buffering the deleterious effects of stress (Levitt et al., 2005).

Stress is broadly considered as a state in which psychosocial and/or environmental demands tax or exceed the adaptive capacity of an individual resulting in psychological and biological changes that could place persons at risk of illness and disease (Cohen, Kessler, & Gordon, 1995), so the potential role of stress and perceived social support in the conceptualization of health and wellbeing by adolescents cannot be underestimated. Also, stress is known to negatively affect health by promoting maladaptive behavioural coping responses (Cohen, 2004), such as smoking and drinking (Lindsey, Joe, & Nebbitt, 2010). There is a significant association between certain types of teasing and distress with social support mediating the effects of teasing (Van Dale et al., 2014). Furthermore, insufficient sleep is reported by adolescents to be associated with a higher likelihood of current use of cigarettes, alcohol, sexual activity, feeling sad or hopeless, and physical fighting (McKnight-Eily et al., 2011).

Social support is a multidimensional construct that refers to the emotionally sustaining qualities of relationships—a sense that one is loved, cared for, and listened to (Umberson & Montez, 2010). Social support enhances an individual's ability to cope with stressful life events to promote both mental and physical wellbeing by influencing cognitions, emotions, behaviours, and biological responses (Cohen, 2004; Kawachi & Berkman, 2001; Uchino, 2004). The stress-buffering and main-effect models are two alternative conceptual models explaining how social support may influence health and wellbeing. The stress-buffering model posits that social support is related to health and wellbeing only when individuals are experiencing stressful life situations (Kawachi & Berkman, 2001). The main-effect model, on the other hand, posits that social support in the form of social resources has a beneficial effect on health and wellbeing regardless of whether or not individuals are experiencing stress (Cohen, Gottlieb, & Underwood, 2000). For example, individuals in social networks may be subjected to social controls and peer pressure that influence normative health behaviours and outcomes, and also the kind of social networks and relationships individuals have may influence whether or not they exercise, eat low-fat diets, consume alcohol, smoke, or engage in drug use/abuse (Robbins, Stommel, & Hamel, 2008).

Social support has a positive effect on adolescents’ health and wellbeing (e.g., Berkman & Lochner, 2002) and parental support in the form of encouragement, in particular, is important for healthy eating and physical activity (Bauer, Laska, Fulkerson, & Neumark-Sztainer, 2011). When adolescents have more psychological autonomy and less psychological control from parents, they experience fewer depressive symptoms (Sher-Censor, Parke, & Coltrane, 2011). Also, subjective wellbeing, self-esteem, frequency of meeting friends, and feelings of not having enough friends, among others, are associated with psychosocial functioning of adolescents (Derdikman-Eiron et al., 2011).

Although a supportive network contributes positively to adolescent health, it may also have a dark side. For example, harassment has been reported to have a significant association with poor self-esteem, depressive symptoms, low body satisfaction, substance use, and self-harm behaviours (Bucchianeri, Eisenberg, Wall, Piran, & Neumark-Sztainer, 2014). Nonetheless, as has been discussed above, social support may work as a potent buffer to mitigate the deleterious effects of stress on health and wellbeing.

Information on the meaning adolescents attach to their health and welling is valuable in making assessment of their health status and outlining the development of indicators of health status that may be used to help judge the need for justified health services and policies (Ford, Davenport, Meier, & McRee 2011). However, there is a paucity of previous research exploring the general health and wellbeing of Ghanaian adolescents from a psychosocial perspective, particularly regarding both social support and stress. Most of what is known about the psychosocial context of adolescent health and wellbeing is based on adolescent samples in western countries. Meanwhile, there are wide international variations in almost all aspects of adolescent health, with young people in sub-Saharan Africa having the poorest health profiles (Patton et al., 2012). Again, malaria, an infectious disease which is practically non-existent in developed countries, accounts for at least 20% of child deaths, 40% of admissions of children to hospital, and more than 50% of out-patient attendance in Ghana (GNMCP, 2006).

The aim of this study, therefore, is to explore the role of perceived social support and stress in the conceptualization and meaning Ghanaian adolescents attach to their health and wellbeing. This seeks to examine how positive interpersonal relationships and support are used as an effective coping resource in times of stress in order to derive a meaningful, qualitative, and holistic representation of health and wellbeing by Ghanaian adolescents.

## Research method

### Design and participants

This qualitative study involved semi-structured interviews. Four senior high schools in Accra, Ghana were randomly selected for the study. First-year students could not participate because they had not reported to school at the time of the study. A purposive sampling technique, by sex, was then used to select 11 participants (five boys and six girls) in the second- and third-year across the four schools. These 11 respondents, with an average age of 16.86 years, were selected from a random sample in a larger mixed methods study constituting 770 males and females. Participants were selected manually after they had completed the quantitative survey. It has been posited that studies that use more than one method require fewer participants, as do studies that use multiple (very in-depth) interviews with the same participants (Lee, Woo, & Mackenzie, 2002).

### Data collection and procedure

In accordance with the ethical guidelines of the APA (2010), initial permission and approval was obtained from the heads of the participating schools. As schools in Ghana do not have a specifically nominated ethics committee responsible for research ethics clearance, school authorities including a school counsellor/chaplain confer on issues about confidentiality, anonymity, and harm to participants before they give permission for their schools to participate. Subsequently, ethical approval was obtained from the University of Essex, UK (FEC 08/04/09). Participants were asked to sign a consent form and those below the age of 18 were given an additional parental consent form for their parent or guardian to sign. Those in boarding school had their house master or mistress sign consent forms in their role as guardians. Participants and school authorities were briefed about the purpose and significance of the study and were assured of their confidentiality and anonymity in any written report.

Interviews, which lasted not more than 1 h, were conducted one-on-one with participants in a classroom, dining hall, or staff common room depending on which one was available and this was captured with a tape recorder . Confirmation of consent was given verbally by each participant to the researchers and their teachers or school authorities before the interviews were conducted. The semi-structured questions on the interview schedule included broad and open questions asking participants: “How close are you to your family, friends, and teachers? Do you get any benefit from them?,” “Do you usually have disagreement and arguments with your family, friends, or teachers?—Why?,” “What makes you feel you are healthy or unhealthy? How or why?,” and “How do you cope with problems or issues you have with your family, friends, and teachers?” Probing questions followed depending on the responses given.

Respondents were reminded that they were not obliged to answer any question that made them feel uncomfortable, especially when it was related to issues on privacy. Accordingly, no respondent reported being distressed and none withdrew from the interviews. If any respondent had become distressed during interviewing, the contingency measure in place was to invite school counsellors to assist in resolving such issues. Data collected has been used for research purposes only. No information or data has been provided that could potentially identify or trace back to the schools or students who participated in the study.

### Data analysis

The audio-recorded interviews were transcribed verbatim for thematic analysis. All audible utterances were transcribed including repetitions of words, pauses in speech, laughter, and sounds accompanying speech such as snapping fingers or hitting the desk top for emphasis.

Each participant's experiences and voices were encouraged through active listening and empathic conversation. Thematic analysis aims to identify, analyse, and report patterns and/or themes by minimizing and describing data in rich detail—where patterns are identified as socially produced (Braun & Clarke, 2006). Thematic analysis also uses a robust, systematic framework for coding data, and for using that coding to identify patterns across the dataset in relation to the research questions (Braun & Clarke, 2014).

Interview transcripts were entered into MAX QDA 10 software to facilitate thematic coding. Given that the sample size is relatively small, the researcher and assistants read all interview transcripts in their entirety to begin the analysis. Research assistants also served as data coders to ensure that there was inter-rater agreement on all codes identified from emerging themes. The broad research questions that formed the basis for the analyses were (1) How do adolescents construe and construct social support and stress? and (2) How does the construction of social support and stress influence the meaning and construction of health and wellbeing? This suggested and necessitated the identification of *a priori* themes by way of deductive coding. By reading and interacting independently with the transcripts with the aim of formulating themes that would confirm to our initial understanding of participants’ responses, the researcher and two assistants delineated *a priori* analytic themes of “social support,” “stress,” and “health and wellbeing.”

The *a priori* and deductively coded themes and their definitions were then entered into MAX QDA 10 and the researcher and assistants separately defined categories and subcategories that referred to specific dimensions or aspects of the three themes. This was then followed by open or inductive coding which involved creating subcategories. Open coding continued until a point where no new properties, dimensions, interactions, and consequences could be identified during the process of coding. For example, one of the categories under the theme “social support” was “advice and encouragement” and a related subcategory was “spirituality or religiosity.” Under the theme “stress,” one of the categories was, “strictness,” and a related subcategory was “inability to express views.” The theme “health and wellbeing” had “ability to perform daily functions” as one of the categories and “ability to take critical decisions” as a related subcategory.

The analysis was an iterative process of going back to the data and the analytical memos to clarify and refine the data and analysis. Through the process of continued linking of the data categories, it became apparent that the categories were not distinct, but that they were underlying similar meanings in the words and expressions of the adolescents, common in all the categories. There were no preconceived ideas about participants; rather, their dialogues, our observations, and analytical memos provided the data for analysis.

### Validity and trustworthiness

Measures were put in place to ensure the validity and trustworthiness of the study (Guba & Lincoln, 1994). With respect to the credibility of this study, a reasonable amount of time was committed to building trust and rapport with the participants before the actual interview commenced. There has been a provision of an authentic account of the participants’ experiences and being transparent about procedures of the data collection and a reflexive view of looking at the entire research process was done out by questioning and checking the process repeatedly to ensure the procedures used were credible. For the sake of transferability of the study, a vivid description of the entire qualitative research process has been done including clarifying issues on validity, trustworthiness, and analysis process. Particularly, there is a succinct description of participants’ experiences, emotions, and coping strategies relating to the responses they gave on the three main thematic variables as well as presenting samples of citations of the respondents for each subtheme.

To ensure that this study was dependable, accounts of the data and data analysis strategy and procedures have been documented. In addition, a “methodological consultancy” (Seale, 2000) procedure was followed by negotiating and agreeing on the adequacy of the processes used in coding, analysing, and interpreting the data by the researcher and assistants. In relation to the confirmability of this study, an attempt was made to let the participants’ voices rise above that of the researcher and research assistants and the conclusions drawn are based solely on the data gathered. These conclusions are examined in relation to other similar empirical studies in order to put it in the right perspective. Furthermore, there has been a reflection on the limitations of this study. In spite of all these, consideration was given to constraints and practicality of the research process as well as what procedures worked best, with due cognisance to the research setting.

## Findings

Through systematic analysis of data, I present salient findings showing how adolescents construct and construe their health and wellbeing and how their construction and meaning of social support and stress influence the construction of their health and wellbeing. Below, the *a priori* themes have been presented with typical excerpts as support where relevant. Pseudonyms have been used to represent the participants, for purposes of anonymity.

### Health and wellbeing

Health and wellbeing was generally understood as ability to perform daily functions, Malaria, and inadequate sleep.

Essentially, ability to perform daily functions included the perception or feeling of *mental strength, ability to take critical decisions* regarding class attendance, *and having a general sense of vitality and strength*. A third-year male student, William, described this feeling of health and wellbeing as “… I know I'm a strong guy mentally … everything—I'm strong and healthy. I know sometimes I don't find it hard to take certain decisions … like going to class.” A third-year female student, Leticia, also said that “I'm in good health and it's helping me—with good health I can study and go about my normal duties in school without problems.” The feeling of mental strength was also expressed as the ability to have a retentive memory and this is supported and described by a third-year male student:

… When I'm not all that feeling well, I can't learn and sometimes too I tend to forget things easily. So anything I learn I have to go over it several times before getting it and if I don't get the chance to do that then everything goes off. (George)

Health and wellbeing was also construed to mean being able to do what one is supposed to, particularly in relation to engaging in sporting activities and other daily life activities, as explained by a third-year female student, Leticia “I am able to do what I'm supposed to do, like sporting activities and other things—I'm able to do it.” This is buttressed by what William said that “sometimes when I feel like I'm not well or too dull, I do some push-ups, about 30—on Saturdays too I go out to play basketball or football and this makes me feel good.”

*Malaria* was construed as major element in the construction of health and wellbeing. For adolescents, a feeling of poor health was largely attributable to malaria. For example, a third-year male student, George, said that “I don't think there should be anything like illness apart from malaria.” Participants were concerned about contracting malaria because it has a negative effect on their health and happens to be the most frequent health problem they have:

… If there should be any sort of health problem that will be malaria. So far that is the only frequent health problem I have. (William)

A second-year male student explained that studying at night in a place with mosquitoes was the main source of getting malaria and contracting malaria means suffering the consequences of the symptoms of malaria such as headaches and stomach aches:

… learning at places where there are a lot of mosquitoes … that is … staying outside when you are supposed to be in, you are likely to be affected by malaria. So these are the diseases that disturb me, malaria—headache and stomach aches. (John)

Finally, *inadequate sleep* at night was said to be a major constituent of health and wellbeing. Inadequate sleep essentially came about as a result of not sleeping early and/or waking up too early which is believed to have a negative effect on wellbeing, including feeling dizzy, headaches, and tiredness during the day. While Agnes said that “… I wasn't resting sometimes … I will go to sleep around 10 pm and wake up around 4 am and I will start my activities … so lack of sleep or lack of rest affects my health,” Eunice, a third-year female student also said that “… Sometimes if I do not get enough sleep or do not sleep early or do not wake up early I feel dizzy during the day.” Mary put it simply as “I usually get headaches if I don't sleep early.” Inadequate sleep, thus, appears to be crucial in adolescents’ construction of their health and wellbeing.

### Social support, health and wellbeing

Social support meant *advice and encouragement* from supportive others. Adolescents receive various kinds of advice from parents when they are going wayward, friend choices, interpersonal relationships with friends, and putting up good behaviours and having manners, all of which have the tendency to enhance mental and social wellbeing. This may also include advice not to stay out late at night to avoid being bitten by mosquitoes and sleeping early enough in order to get adequate rest. A third-year female student expressed the advice she receives from her parents:

… They [parents] are able to advise me, they tell me when I am going wrong, they are able to correct me when I'm going wrong, they tell me what is right, to know the kind of friends to choose, they tell me what to do when I come to school, the way I talk with my friends and others. (Eunice)

Mothers, especially, gave advice on ways of reconciling and amicably settling disagreements, quarrels adolescents had with their friends and others. A second-year female student exemplified this feeling of support:

… ok sometimes when I'm in a situation with my friends—for instance we've quarrelled and we are not on the same terms, I go to my mum and ask her how am I supposed to reconcile with my friends and then she tells me the way and how to go about it. (Agnes)

Advice and encouragement from fathers was also regarded as a useful source of support for health promotion behaviours. A third-year female student explained how her father advised her about the negative effects of consuming too much sugar, salt, and fats as well as controlling her weight:

… Ok my Dad, he likes talking about intake of sugar, salt and others. He always talks about those kinds of food. He has always been talking about me being big so I shouldn't eat a lot of oil. (Mary)

Eunice, added that “… they [parents] tell me the kind of food to eat and the right time to eat.” Advice and encouragement by friends was also a useful source of support for engaging in health promotion behaviours, especially in relation to eating healthy food. For example, Mary said “… when I'm going to buy food and the food does not look healthy they [friends] will talk and discourage me from buying”.

Besides advice and encouragement, *religiosity or spirituality* was described as a key element in the construction of social support, with respect to health and wellbeing. In this particular context, social support was construed as being spiritual (i.e., praying, going to church, and meeting spiritual leaders). When feeling sick or ill, adolescents resorted to prayer and going to church as a means to get well and feel better. In Agnes's own words “… when I am sick my mum takes extra care of me, she will leave everything that she is doing if it is prayer that she needs to pray she will take me to the church to pray.”

Spiritual or religious faith as a form of social support was also believed to enhance health or restore wellbeing through interactions and sharing problems with spiritual leaders. A healthy talk with a pastor was spiritually uplifting and was described to be emotionally rewarding. According to William:

… Most at times when I encounter problems [illness or sickness], I consult my spiritual leaders and we have a healthy talk and very soon I forget everything [emotional problems].

With regard to health promotion behaviours, alcohol intake particularly, it was felt that being spiritual or religious made alcohol consumption unappealing, apparently because this behaviour is thought to be unacceptable. John, a second-year male student exemplified this feeling by saying that “Ok … I have drunk alcohol before, but since I became a Christian I've not touched any alcohol.” This highlights the crucial role of spiritual and religious beliefs as a major source of social support in health and wellbeing.

### Stress, health, and wellbeing

Participants felt that *strictness*, *teasing, arguments, and quarrelling* emerging from their relationships with family, friends, and teachers were the major source of stress they encountered.

Strictness, as a construction of stress, essentially emanated from the inability to express points of views and being dictated to. Adolescents felt that their views and opinions appeared not to matter to parents and that they were not allowed to leave home and not given the freedom to explore life, thereby affecting the social wellbeing. John gave a vivid description of this feeling by saying that “… It is like we are in the same house or something but on certain issues I can't discuss with my parents, most at times because they are not all that open, especially my dad, that's how it is … It's like sometimes they give me their ideas so I also want them to take my ideas because sometimes I want to explore and not locked up in the house and those things. I don't go out often but I like to go out.” Dorcas, a third-year female student added that “… my dad is sometimes very strict and somehow too he is a free type but when that strictness of his comes, he is very hard to stand.” Furthermore, strictness by parents appeared to have a negative effect on confidence. For instance, John said that “… because I'm not able to tell them certain things that hurt me or I have not gone to that extent of telling them I might be facing certain things as an adolescent—but the relationship I have with them cannot give me that confidence to tell them certain things.” This notwithstanding, strictness by parents to let their children stay home most of the time, especially at night, may also prevent malaria as a result of mosquito bites and also have adequate sleep to prevent day time sleepiness and tiredness.

In addition, strictness for adolescents, took the form of fear of teachers as a result of not being able to express themselves which made their peers tease and laugh at them. For example Mary said that “… sometime some of the words they [teachers] use are … it pierces [hurts]—words like ‘I have no hope in you’ and all those stuff, sometimes it pierces and I get disappointed.” This is further encapsulated in the following quote:

Some of the teachers are too strict that when you see them you become afraid and that makes me have some headaches, I don't feel normal especially in the subject he/she teaches. (Eunice)

Teasing as an element of the construction of stress bordered on not being shown respect by peers and this leads to being laughed at, which subsequently affects the ability to maintain one's concentration. This was said by a second-year female student, Esther:

… because you may feel bad when you're talking and someone [friend/s] just shut you up like that and others will be laughing and teasing you. It feels very bad and sometimes I cannot even concentrate.

Argument and quarrelling with friends also appeared to embody the construction of stress. This source of stress emerged when adolescents quarrel or argue with their friends and it leads to them not talking to each other. This made them feel isolated and made them become very emotional and irritated. In the words of Esther:

When my friends are not talking to me … I mean a particular friend not any other friend, is not talking to me, I feel very bad, I think about it and sometimes loose appetite because it's like she is my closest friend so if she's not talking to me I wonder why. It makes me feel like not talking and I insult whoever tries to talk to me.

Arguments and quarrels are not peculiar to only friends, adolescents sometimes argued and quarrelled with their mothers as well. According to Mary, “… like maybe I will be there she will be calling me and I didn't hear it, I didn't hear her well and later she will be shouting at me and I will ask her why she is shouting, she will tell me that she called me and I didn't mind her and this makes me sad and feel like leaving the house.”

In a sense, from the foregoing, it appears that the relationships adolescent have with significant others were not always supportive. Lack of or inappropriate support appeared to be a major source of stress. For example, William said that “emotionally I will say I'm not all that healthy because like the kind of love that I want, I don't get it because they [parents] are always at work and they are travelling—they will travel leaving me and my brother, my mother too will be going to work. So it is like the kind of attention I want from them, I don't get it, which is affecting me.” Thus, it could be suggested that where social support is regarded as beneficial and positive, there is a low feeling of stress.

## Discussion

The aim of this study was to explore the role of perceived social support and stress in the meaning adolescents attach to their health and wellbeing. The results pointed to the fact that ability to perform daily functions was a key theme that represented the meaning of health and wellbeing and this was influenced by (1) social support—encouragement and advice, religiosity or spirituality, and (2) stress—strictness, teasing, quarrels, and arguments.

The results support findings of previous studies in that adolescents attribute their health and wellbeing to their inability to function well psychosocially (Derdikman-Eiron et al., 2011). Ability to perform daily functions was in turn influenced by inadequate sleep and resulting in poor physical and psychological functioning. For example, insufficient sleep has been found to be associated with the use of cigarettes, alcohol, and feeling sad or hopeless (McKnight-Eily et al., 2011).

Social support was in the form of encouragement and advice. This finding is similar to what has been found in previous studies that parental support in the form of encouragement is important for healthy eating and physical activity (Bauer et al., 2011). Also, it has been found that parental support could contribute to a negative construction of health and wellbeing with respect to strictness and harassment, and this is consistent with the findings of previous studies. For example, when adolescents’ have a perception of more promotion of psychological autonomy and less psychological control from parents they experience fewer depressive symptoms (Sher-Censor et al., 2011). Also, teasing by peers has a significant association with distress with social support mediating teasing and some externalized outcomes (Van Dale et al., 2014). This goes to suggest the importance of peer relations in promoting and sustaining the quality of life of adolescents (Helseth & Misvær, 2010).

Adolescents felt that arguments and quarrels were the main source of stress that impacted negatively on their health and wellbeing. These arguments and quarrels pose a major source of stress if not managed properly. Previous studies have found that culturally-universal stressors such as parent–child disagreement substantially influenced depressive symptoms (Stein, Gonzalez, & Huq, 2012). Also, Glick, Rose, Swenson, and Waller (2013) report that adolescents whose mothers’ friendships are characterized by conflict and antagonism are more likely to have friendships that are high in negative friendship qualities as well as elevated internalizing symptoms. In tandem with arguments and quarrels, strictness by significant others was also felt to be stressful. However, this strictness may compel adolescents to stay indoors, especially at night to prevent mosquito bites and malaria and also be able to have adequate sleep at night. It has been posited that parental monitoring, supervision, and high quality of parent-child relationship dissuade involvement in high-risk behaviour, and authoritative parenting generally leads to the best outcomes for adolescents (DeVore & Ginsburg, 2005).

Finally, religiosity or spirituality was an effective resource of social support that helped in the mitigation of distress and enhancement of health and wellbeing. Congruent to what has been found in previous studies, religion and spirituality have been found to provide systems of meaning and feelings of strength to cope with stress and adversity (Hill & Pargament, 2003). Adolescents in the present study said they quit drinking alcohol as soon as they became spiritual, and this is in accord with findings that indicate that adolescent spirituality is a protective factor against deleterious alcohol use (Delva, Andrade, Sanhueza, & Han, 2013). Furthermore, religious or spiritual characteristics have shown to have strong relationships with depression (Dew et al., 2010). The culture in Africa and in Ghana, particularly, may have been the underlying factor in adolescent religiosity and spirituality. Religion is a fundamental, perhaps the most important, influence in the life of most Africans (Awolalu, 1976), which may not be the same in western cultures. From a cultural perspective, therefore, religion or spirituality may have had a substantial influence in the construction and meaning of social support, stress, and health and wellbeing in this study.

Thus, it is important for significant others (i.e., family, peers, teachers and, spiritual leaders) to play meaningful roles in the lives of Ghanaian adolescents through encouragement, advice, and mutual respect. Adolescents could be advised and encouraged to engage in physical activity, good eating habits, sleeping in mosquito nets, keeping surroundings clean, and having adequate sleep. From a critical analysis of the interview transcripts and findings of the study, it appears that female students reported seeking more support from significant others, suggesting that they would have experienced more stress and therefore lower health and wellbeing. For adolescents who are religious or spiritual, their leaders could encourage them to frequent places of worship for prayers and spiritual upliftment and other life fulfilling activities. These meaningful roles could serve as mitigating factors in stress experience, particularly in the midst of arguments, quarrels, teasing, and when significant others are being overly strict. These would eventually lead to the capacity and ability to overcome stressful life events for effective performance of daily life functions—health and wellbeing.

## Limitations

As this study was qualitative in nature and used a relatively small sample size, it could be a potential threat to the study's trustworthiness and generalization to the rest of the population from which this sample was drawn, and so generalizations should be done with caution. Perhaps, a larger sample size would have discovered much more representative views and opinions. This notwithstanding, as the study was part of a broader mixed methods study, respondents had earlier provided a large amount of quantitative data so the interviews could not go in-depth. Also, because this was part of a postgraduate research study, it was constrained by time and financial resources. Nonetheless, the use of probing questions subsequent to each interview question appears to have absorbed the apparently limited sample size. Finally, it is worth noting that thematic analysis has limited interpretative power beyond mere description if it is not used within an existing theoretical framework that anchors the analytic claims that are made (Braun & Clark, 2006).

## Conclusion

This study has demonstrated that the psychosocial context of adolescent development is important in the meaning they attach to their health and wellbeing. Particularly, social support is crucial in mitigating the negative effects of stress on health and wellbeing. Knowledge about the meaning of health and wellbeing to adolescents provides an opportunity to devise strategies that would enhance social support to mitigate the adverse effects of stress to consequently promote the health and wellbeing of Ghanaian adolescents.
